# Applications of cluster analysis to the creation of perfectionism profiles: a comparison of two clustering approaches

**DOI:** 10.3389/fpsyg.2014.00343

**Published:** 2014-04-23

**Authors:** Jocelyn H. Bolin, Julianne M. Edwards, W. Holmes Finch, Jerrell C. Cassady

**Affiliations:** Department of Educational Psychology, Ball State UniversityMuncie, IN, USA

**Keywords:** fuzzy clustering, k means clustering, classification, perfectionism, profiles

## Abstract

Although traditional clustering methods (e.g., K-means) have been shown to be useful in the social sciences it is often difficult for such methods to handle situations where clusters in the population overlap or are ambiguous. Fuzzy clustering, a method already recognized in many disciplines, provides a more flexible alternative to these traditional clustering methods. Fuzzy clustering differs from other traditional clustering methods in that it allows for a case to belong to multiple clusters simultaneously. Unfortunately, fuzzy clustering techniques remain relatively unused in the social and behavioral sciences. The purpose of this paper is to introduce fuzzy clustering to these audiences who are currently relatively unfamiliar with the technique. In order to demonstrate the advantages associated with this method, cluster solutions of a common perfectionism measure were created using both fuzzy clustering and K-means clustering, and the results compared. Results of these analyses reveal that different cluster solutions are found by the two methods, and the similarity between the different clustering solutions depends on the amount of cluster overlap allowed for in fuzzy clustering.

## Introduction

Clustering is a common method used in the psychological, social, and physical sciences to identify subgroups or profiles of individuals within the larger population who share similar patterns on a set of variables. Traditional methods of clustering (e.g., K-means) attempt to place each individual case into a cluster with other observations with which it shares a similar score pattern (Everitt et al., 2011). Such traditional hard clustering methods allow an individual to belong to only one cluster. Such an approach also ignores the fact that an individual may share traits with multiple subgroups in the population, and thus potentially belong to more than one such cluster. The purpose of this study is to showcase the use of a soft clustering technique, fuzzy clustering, that is currently underutilized in the social sciences. Unlike traditional hard clustering methods, fuzzy clustering allows for individual cases to simultaneously belong to more than one cluster, thus having the potential to inform not only the cluster with which a case has the strongest membership but also how each case is related to each of the clusters (Everitt et al., 2011). As a result, fuzzy clustering can provide the researcher with a more realistic picture of subgroups and subgroup relations within the population. Rather than assuming that an individual is only a member of a single subgroup, allowing the individual to share membership in multiple clusters reflects the reality that such membership does not need to be an either/or proposition (Gan et al., 2007). Thus, fuzzy clustering has the potential to provide more information about the structure of the data than other clustering methods (Kaufman and Rousseeuw, 2005).

This paper provides a comparison of clustering solutions based on the traditional K-means and fuzzy clustering approaches using the same data set in order to demonstrate the similarities and differences between the techniques and showcase the utility of the unique features associated with fuzzy clustering. This comparison will be done using a data set measuring aspects of perfectionism in a college undergraduate sample. The perfectionism data was chosen both to appeal to the intended social science audience for this study as well as to help add to the growing discussion of a group based perfectionism orientation. The following sections provide a description of the data and research question this data set was attempting to answer.

### The field of perfectionism and need for clustering research

Perfectionism is generally defined as a condition in which the individual holds excessively high personal standards with a tendency toward overly critical review of personal achievements and behaviors (Stoeber et al., 2009). Originally viewed as a singular dimension that was deleterious to optimal functioning, Hamachek (1978) introduced a line of inquiry that has dominated perfectionism research in the past 35 years identifying both “normal” and “neurotic” perfectionism. Since the 1990's, there has been universal agreement that perfectionism is a multidimensional construct, with multiple measures constructed to assess these factors, including the Multidimensional Perfectionism Scale (Hewitt and Flett, 1991), Almost Perfect Scale (Slaney et al., 2001), and the Frost Multidimensional Perfectionism Scale (FMPS) (Frost et al., 1990).

### Multidimensional orientation of perfectionism

While the items and eventual factor structure for each scale differ, the underlying conclusions of the research in the field confirms essentially similar patterns of responses, with both positive (e.g., high personal standards, organization) and negative aspects (e.g., elevated self-criticism, susceptibility to external pressures) of perfectionism being identified (e.g., Stoeber and Otto, 2006). The FMPS has been perhaps the most commonly studied set of perfectionism items and originally identified a six-factor solution to the 35-item scale. While several studies have used the FMPS and provided strong validation for the scale and a multidimensional nature for perfectionism, there have been multiple alternative representations for the construct (Stoeber, 1998; Purdon et al., 1999; Harvey et al., 2004). The various factor solutions for the FMPS provide ample opportunity to analyze a pattern of performances in the normal population. However, in a systematic comparison of the factorial representations of the FMPS, Harvey et al. (2004) provided compelling evidence that their four-factor solution was durable, explained the variance effectively and captured the representations offered by other research teams. Their reconceptualization of the 35-item scale produced the following four factors (a) Negative Projections—items addressing the tendency to make social comparisons and hold self-doubt over competence; (b) Achievement Expectations—items addressing holding high personal standards and ego involvement goal orientation; (c) Parental Influences—items addressing parental influences and reactions to performance; and (d) Organization—consistently identified in other factor solutions for the FMPS that identify tendencies toward organization and neatness. Their analysis for this new factor structure showed theoretical similarity to Stoeber's (1998) four-factor structure, but demonstrated a better fit to the data and strong construct validity with the original six-factor solution (Frost et al., 1990) upon which the scale was created.

### Group-based orientation

An alternative approach to examining perfectionism in learners has been to adopt a group-based or individualistic orientation, where the focus is on constructing perfectionism profiles based on responses to one of the primary assessment tools (Stoeber and Otto, 2006). The predominant approach to reviewing perfectionism through a group-based orientation has been to use cluster analysis to generate the profiles of perfectionism identified in the response data (e.g., Parker, 1997; Rice and Dellwo, 2002; Grzegorek et al., 2004; Ashby and Bruner, 2005; Gilman et al., 2005; Mobley et al., 2005). As with the multidimensional orientation, research into the group-based view of perfectionism has generated several alternative conceptualizations for “types” of perfectionism (e.g., Parker, 1997; Rice and Dellwo, 2002; Grzegorek et al., 2004; Ashby and Bruner, 2005; Gilman et al., 2005; Mobley et al., 2005). Stoeber and Otto's (2006) review of the extant research revealed the bulk of group-based perfectionism research can be summarized rather effectively by reviewing the presence of two dimensions of perfectionism: evaluative concerns and personal standards. In their proposed tripartite framework to explain the various research, non-perfectionists were identified as those with low levels of personal standards perfectionism (regardless of evaluative concerns). For those with high levels of personal standards, individuals with low evaluative concerns were classified as “healthy perfectionists” and those with high evaluative concerns were classified as “unhealthy perfectionists.” Gaudreau and Thompson (2010) proposed an alternative model based on this same framework, suggesting that the tripartite framework may be an incomplete representation of dispositional perfectionism. In particular, Gaudreau and Thompson (2010) proposed a 2 × 2 model—identifying individuals who were (a) non-perfectionists, (b) pure personal striving perfectionists, (c) pure evaluative concerns perfectionists, and (d) a “mixed” perfectionist who holds both high personal standards and evaluative concerns. The difference in these two models is the addition in the 2 × 2 model of the group of perfectionists with only personal standards perfectionism (no evaluative concerns).

Two key questions arise when reviewing the debate regarding the Gaudreau and Thompson (2010) and Stoeber and Otto (2006) representations for dispositional perfectionism. The first is whether the individuals with characteristically low levels of personal standards perfectionism can be split into two groups (Gaudreau, 2013). The second is a fundamental issue of whether each cluster is a distinct group with clear differentiation. That is, in both models there is the typical assumption that the separate clusters do not overlap, capturing distinct representations of “types of perfectionists.” This study takes on both of these questions by using perfectionism data to compare two different clustering approaches and showcase the potential benefits of the fuzzy clustering approach while also attempting to add to the perfectionism profile literature.

### Clustering methods

As demonstrated above, research into group-based orientation is commonly assessed using K-means clustering. While this clustering method has been shown to be useful and effective it does not allow researchers to account for overlap among the clusters. In order to address the issue of overlap, we propose the use of fuzzy clustering. The following section provides descriptions of both the K-means and fuzzy clustering algorithms, highlighting their similarities and differences.

### K-means clustering

K-means clustering is a common centroid based clustering method that identifies a specified number of non-overlapping clusters within data (Gan et al., 2007). It requires the researcher to pre-specify the number of clusters and then places each individual into one of them. It should be noted that the actual profile (i.e., means on the variables used to cluster) of the clusters is not pre-specified, but only the number. The K-means clustering algorithm is based on the following steps.

1. The researcher indicates the number of clusters.
2. Initial cluster centroids are formed either by using random selection for the K clusters, or through pre-specification of cluster centroids by the researcher.
3. The squared Euclidean distance (ESS) is calculated based on the current cluster solution.
4. Each individual is reassigned to the cluster to whose centroid it is closest.
5. The cluster centroids are updated after each reassignment.
6. Steps 3–5 are repeated until no further reassignment of individuals to clusters takes place, i.e., each individual is in the cluster with the nearest centroid.

ESS is expressed as (Izenman, 2008):

(1) $$ESS=\sum_{k\;=\;1}^{K}\sum_{c(i)\;=\;k}{\left(x_{i}-{\bar{x}}_{k}\right)}^{\prime }\left(x_{i}-{\bar{x}}_{k}\right)$$

where *K* is the number of pre-specified clusters, *x*_*k*_ is the centroid for cluster *k*, *x*_*i*_ is a vector of scores on the variables used to cluster individual *i*, and *c*(*i*) is the cluster containing the individual. The *ESS* is calculated for each iteration of the process described above, until all reassignments are completed, and *ESS* itself is minimized. When such convergence is reached, the researcher then examines the resultant clusters in order to determine whether they are substantively meaningful and clearly distinct based upon the pattern of means on the variables used to cluster, as well as other variables that are hypothesized to differ among the clusters. By definition this latter step in the clustering process involves subjective judgment on the part of the researcher.

### Fuzzy clustering

Fuzzy clustering is an extension of the traditional K-means algorithm. However, unlike K-means clustering, fuzzy clustering focuses on cluster membership based on fuzzy set theory (Everitt et al., 2011). Given this paradigm, fuzzy clustering allows individuals to have multiple cluster memberships, thereby providing useful information about the degree of cluster overlap in the population, as well as information about the relative membership of each individual within each cluster. Thus, in fuzzy clustering each case is allowed (but not required) to have partial membership in multiple clusters. For example, cluster membership for a hypothetical case might exhibit the following pattern: the individual has a 56% membership share in cluster 1, a 32% share in cluster 2, and a 12% share in cluster 3. As implied in this example, the degree to which a case belongs to a certain cluster is indicated by its membership share, which ranges from 0 to 1 (i.e., it is the proportion of the case that belongs to the cluster; Guldemir and Sengur, 2006). The algorithm for fuzzy clustering is based on minimizing the following objective function, as described by Kaufman and Rousseeuw (1990):

(2) $$F=\sum_{k\;=\;1}^{K}\frac{\sum_{i}\sum_{j}u_{ik}^{2}u_{jk}^{2}d_{ij}}{2\sum_{l}u_{lk}^{2}}$$

Here, *k* is as defined above. In addition, *u*_*ik*_ is a membership coefficient reflecting the membership share for observation *i* in cluster *k*. For a given individual, $\sum_{k\;=\;1}^{\;K}u_{ik}=1$ and all *u*_*ik*_ ≥ 0. The value *d*_*ij*_ is a measure of dissimilarity for observations *i* and *j*, across the variables used in the clustering. For continuous data, the Euclidean distance measure *d*_*ij*_ is expressed as:

(3) $$d_{ij}={\left({\left(x_{i}-{\bar{x}}_{k}\right)}^{\prime }\left(x_{i}-{\bar{x}}_{k}\right)\right)}^{5}$$

Thus, fuzzy clustering makes use of an iterative algorithm in which the function in (2) is minimized through altering the values of *u*_*ik*_. The membership coefficients are in turn calculated as (Kaufman and Rousseeuw, 2005):

(4) $$u_{ik}=\frac{1}{\sum_{k^{\prime }}^{K}{\left(\frac{d_{ik}}{d_{ik^{\prime }}}\right)}^{2(m\;-\;1)}}$$

In (4), *d*_*ik*_ and *d*_*ik*'_ represent the distances between observation *i* and clusters *k*, and *k*' (*k* ≠ *k*′), and *m* is the membership exponent, which will be described in detail below.

In the context of fuzzy clustering, the amount of overlap among clusters across the sample is referred to as the degree of fuzziness. The degree of fuzziness allowed in a particular analysis can be controlled by the researcher through manipulation of a quantity known as the *membership exponent* (*ME*). This value ranges from 1 (minimal fuzziness and equal to K-means) to infinity, where larger values are associated with a greater degree of fuzziness (Gan et al., 2007). Previous studies have recommended setting the membership exponent to 2 in many applications in practice (Lekova, 2010; Maharaj and D'Urso, 2011). The membership exponent chosen by the researcher will depend on how much cluster overlap the researcher expects in their data.

### Prior research applications of fuzzy clustering

Researchers in fields such as medicine, technology (e.g., imagery software, computer science), and business already use fuzzy clustering with some regularity. Specifically, fuzzy clustering has been used in gene research for cancer prediction (Alshalalfah and Alhajj, 2009), tumor classification (Wang et al., 2003), research with MRI data (Ahmed et al., 2002), changes in remote sensing images (Ghosh et al., 2011), satellite image retrieval (Ooi and Lim, 2006), bankruptcy forecasting (De Andrés et al., 2011), computer grading of fish products (Hu et al., 1998), and classification of management styles (Andrews and Beynon, 2011).

Several studies using existing and simulated data have been conducted to compare the performance of traditional hard clustering methods to fuzzy clustering. Based upon these studies, it appears that fuzzy clustering can be a useful clustering method due to its ability to produce both hard and soft clusters, show the relationship of clusters to one another, and deal effectively with outliers (Goktepe et al., 2005; Grubesic, 2006). The ability to handle outliers is an especially important feature of fuzzy clustering given that outliers can be a serious problem for other clustering algorithms such as K-means (Grubesic, 2006). In the context of fuzzy clustering, the outlier's membership is distributed throughout the clusters, instead of the outlier being placed into one cluster. Unlike fuzzy clustering, K-means clustering would have the outlier belong to one cluster, which can skew the structure of the clusters (Grubesic, 2006). Additionally, fuzzy clustering has been shown to accurately group cases into clusters with real and simulated data (Schreer et al., 1998; Goktepe et al., 2005). Schreer et al. (1998) found that with artificial data both fuzzy clustering and K-means clustering on average misclassified 12% of the data and had similar cluster solutions. While fuzzy clustering has been shown to produce similar clusters to K-means on simulated data, fuzzy clustering was able to show the strength of membership for each cluster as well (Schreer et al., 1998).

Despite the demonstrated benefits, fuzzy clustering has yet to be fully utilized throughout the social and behavioral sciences. It does appear, however, that researchers in the social and behavioral sciences are aware that not all clusters are discrete. For example, in a study of personality types using principal-components analysis, Chapman and Goldberg (2011) describe their case cluster structures as indistinct or “fuzzy,” rather than discrete, when referring to the overlapping of clusters in visual representations of their data. Although graphical representations can be quite informative, it is also important to be able to quantify the degree of such overlap. The utilization of fuzzy clustering could be considered a more natural approach in many applications, because behavioral clusters are not always distinct, and there will be some overlap due to the abstract nature of human behavior.

## Methods

In order to demonstrate the utility of fuzzy clustering, a comparison of traditional K-means clustering and fuzzy clustering was made using a previously analyzed data set from a study on perfectionism. The FMPS (Frost et al., 1990) was used in a sample of undergraduate university students enrolled in educational psychology and business education courses. Data were collected over the course of three academic years, where participation in data collection satisfied a course requirement. Collectively, 486 students (304 females, 182 males) participated in the study. A total of 30 cases had to be deleted due to missing data bringing the final sample size to 456. As only a small number of cases had missing information, simple listwise deletion was used. The average age of the participant was 20.97 (*SD* = 3.3), and the sample was predominately Caucasian (92.6%), consistent with the population from which the sample was recruited.

As mentioned earlier, in a systematic comparison of the factor representations of the FMPS, Harvey et al. (2004) provided compelling evidence in favor of their four-factor solution. These four factors included Negative Projections, Achievement Expectations, Parental Influences and Organization. In order to compare and demonstrate the performance of hard and fuzzy clustering methods, a cluster solution generated by K-means, and a cluster fuzzy clustering of the four FMPS Harvey factors were run using R statistical software, version 2.13.1 (R Development Core Team, 2010). The fanny() function located in the CLUSTER R package was used for fuzzy clustering, and the kmeans() function located in the STATS R package for K-means clustering. For both the fuzzy clustering and K-means solutions, the default R settings were used. By default, the K-means clustering algorithm in R uses the Hartigan-Wong algorithm (Hartigan and Wong, 1979), and for fuzzy clustering R uses a Euclidian dissimilarity measure with a measurement exponent of 2.0. First, the default fuzzy clustering solution was compared to the K-means clustering solution in terms of similarity of cluster structure, cluster solution fit, and cluster interpretation. Following this comparison, the membership exponent for fuzzy clustering was manipulated to demonstrate differences in cluster interpretation between fuzzier and crisper cluster solutions for the same data. To accomplish this comparison, the membership exponent was changed to 1.2 (which is virtually the smallest membership exponent R will allow) to obtain a crisp cluster solution, and the cluster solutions were again compared in terms of similarity of results. The purpose of changing the membership exponents is to show how manipulating the degree of fuzziness can provide different but meaningful cluster solutions.

## Results

### K-means cluster solutions

Descriptive statistics and psychometric information for the FMPS Harvey subscales appear in Table 1. Prior to clustering, multicollinearity was assessed through use of zero order correlations and VIF statistics. Zero order correlations between the Harvey subscales ranged from *r* = 0.032–0.618 with VIF ranging from 1.186 to 1.861. Together, these results indicate that multicollinearity was not a concern, and the clustering proceeded as planned.

**Table 1:** **Descriptive statistics and properties of the FMPS harvey subscales**.

	**# of items**	**Min–max**	**Mean**	**Standard deviation**	**Cronbach alpha**
Negative projections	12	12–60	31.20	8.44	0.86
Ach expectations	8	8–40	28.44	5.35	0.85
Parental influence	9	9–45	24.08	6.86	0.89
Organization	6	6–30	24.00	4.60	0.89

Originally, two different K-means cluster solutions were created: one solution based on the raw subscales and one solution using standardized subscales. Because the FMPS Harvey subscales have differing numbers of items, it was important to ensure that the differential weighting of the variables did not impact the interpretation of the cluster solution. After comparing the standardized and unstandardized solutions, it was determined that both solutions supported the same conceptual profiles, thus the cluster solution based on the unstandardized variables was chosen for ease of interpretation.

As K-means clustering is the standard approach, it was performed first. Initially, however, a hierarchical cluster analysis was performed in order to determine the number of clusters for the K-means approach. Based on the visual information from the dendrogram, three and four cluster solutions were created using K-means cluster analysis. Comparison of the two different K-means solutions revealed that the four-cluster solution was more consistent with the current theoretical models of perfectionism. Cluster means for the four-cluster solution appear in Table 2. Within-cluster R^2^ was calculated for each cluster as a measure of cluster similarity, ranging from 0.69 to 0.80 indicating moderate to high within cluster similarity.

**Table 2:** **Means for the K-means and fuzzy clustering hard cluster solutions**.

	**Neg. Proj**	**Achexp**	**Parinf**	**Org**
	***M* (*SD*)**	***M* (*SD*)**	***M* (*SD*)**	***M* (*SD*)**
**K-MEANS**				
Cluster 1—externalized perfectionists (*n* = 103)	32.78 (3.79)	25.66 (3.72)	25.93 (5.03)	20.62 (4.13)
Cluster 2—mixed perfectionists (*n* = 99)	42.74 (4.84)	32.22 (4.07)	32.50 (5.59)	24.68 (3.97)
Cluster 3—internalized perfectionists (*n* = 121)	30.21 (4.06)	32.25 (3.22)	21.17 (4.19)	27.03 (2.96)
Cluster 4—non-perfectionists (*n* = 133)	22.27 (4.36)	24.31 (4.35)	19.04 (3.78)	23.36 (4.68)
**FUZZY FOUR CLUSTER SOLUTION**				
Cluster 1 (*n* = 92)	33.40 (4.58)	29.33 (5.55)	25.16 (6.34)	23.04 (5.47)
Cluster 2 (*n* = 126)	41.13 (4.84)	31.61 (3.89)	31.10 (5.51)	24.52 (3.78)
Cluster 3 (*n* = 110)	26.41 (6.01)	26.95 (6.04)	20.25 (4.84)	23.49 (5.67)
Cluster 4 (*n* = 128)	23.94 (3.39)	25.95 (3.93)	19.69 (2.81)	24.63 (3.34)
**FUZZY THREE CLUSTER SOLUTION**				
Cluster 1 (*n* = 136)	40.94 (5.23)	31.65 (4.07)	30.88 (5.67)	24.46 (3.92)
Cluster 2 (*n* = 128)	31.38 (4.52)	28.58 (5.78)	23.65 (5.87)	22.74 (5.76)
Cluster 3 (*n* = 192)	24.17 (4.41)	26.07 (4.60)	19.56 (3.59)	24.52 (3.99)
**FUZZY ME 1.2 CLUSTER SOLUTION**				
Cluster 1 (*n* = 110)	32.69 (3.80)	25.67 (3.72)	25.75 (4.97)	20.84 (4.13)
Cluster 2 (*n* = 100)	42.68 (4.85)	32.21 (4.05)	32.43 (5.60)	24.71 (3.96)
Cluster 3 (*n* = 119)	29.90 (4.02)	32.34 (3.19)	20.94 (4.25)	26.93 (3.09)
Cluster 4 (*n* = 127)	22.07 (4.34)	24.20 (4.29)	19.01 (3.74)	23.44 (4.76)

ME, Membership Exponent used for Creation of Fuzzy Clusters. When not specified, default ME of 2 for fuzzy clustering was used.

The clusters listed in Table 2 were tentatively named based on the relationships observed among the four Harvey factors and are described briefly. First, Externalized Perfectionists (K-means cluster 1) were characterized primarily by having low organization and achievement expectations with moderate levels of parental influence and negative projections. The term Externalized Perfectionism was selected as it depicts the profile of an individual with moderately elevated perfectionism, driven primarily by external influences (similar to notions of socially prescribed perfectionism). Second, the Mixed Perfectionists (K-means cluster 2) reported high overall levels of perfectionism, with heightened negative projections, achievement expectations and parental influence, but reported moderate levels of organization. Internalized Perfectionism (K-means cluster 3) included individuals with moderate overall perfectionist tendencies who demonstrated heightened levels of organization and personally-prescribed achievement expectations. Finally, Non-Perfectionists (K-means cluster 4) were those individuals in the sample who did not demonstrate an elevated degree of any of the Harvey perfectionism factors – as such those in the sample with no clear perfectionist tendencies.

### Similarity of K-means and fuzzy clustering solutions

Tables 2, 3 provide information regarding the similarity of the K-means and fuzzy clustering solutions. As already discussed above, Table 2 presents the cluster means for the original 4 cluster K-means solution and the default 4 cluster fuzzy clustering solution. Also presented are a 3 cluster fuzzy clustering solution and the 4 cluster fuzzy clustering solution using a membership exponent of 1.2, which will be discussed in more detail below.

**Table 3:**
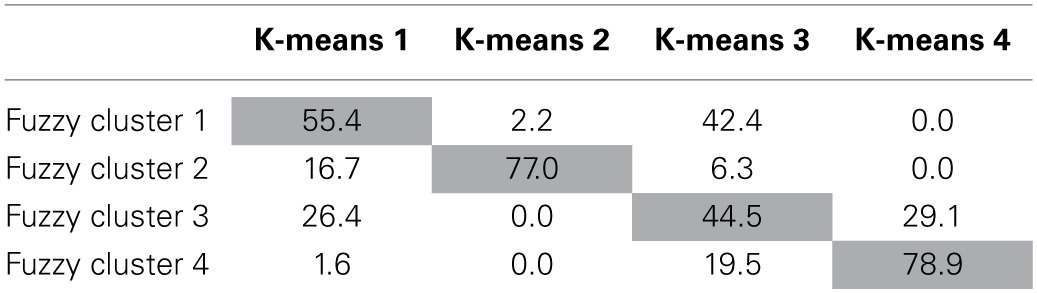
**Percentage of fuzzy cluster solutions that belong to corresponding k-means clustering solutions with a membership exponent of 2.0**.

As can be seen in Table 2, the cluster means for the 4-cluster K-means solution and the 4 cluster fuzzy clustering solution show similar patterns indicating similar cluster interpretation. K-means cluster 1 (externalized perfectionists) and K-means cluster 3 (internalized perfectionists) are related closest to cluster 1 of the 4-cluster fuzzy cluster solution. According to Table 3, fuzzy cluster 1 has the highest percent of participants belonging to the externalized perfectionists as defined by K-means (55.4%), but also has considerable overlap with the internalized perfectionists (42.4%) K-means cluster. The second K-means cluster (mixed-perfectionists) was most closely associated with fuzzy cluster 2. Fuzzy cluster 2 had the highest percent of participants classified by K-means as mixed perfectionists (77.0%) with the second highest percent belonging to externalized perfectionists at only 16.7%. K-means cluster 4 (non-perfectionists) relates most strongly to fuzzy cluster 4, with 78.9% of the cases in this cluster belonging to the K-means non-perfectionism cluster.

**Figure 1:**
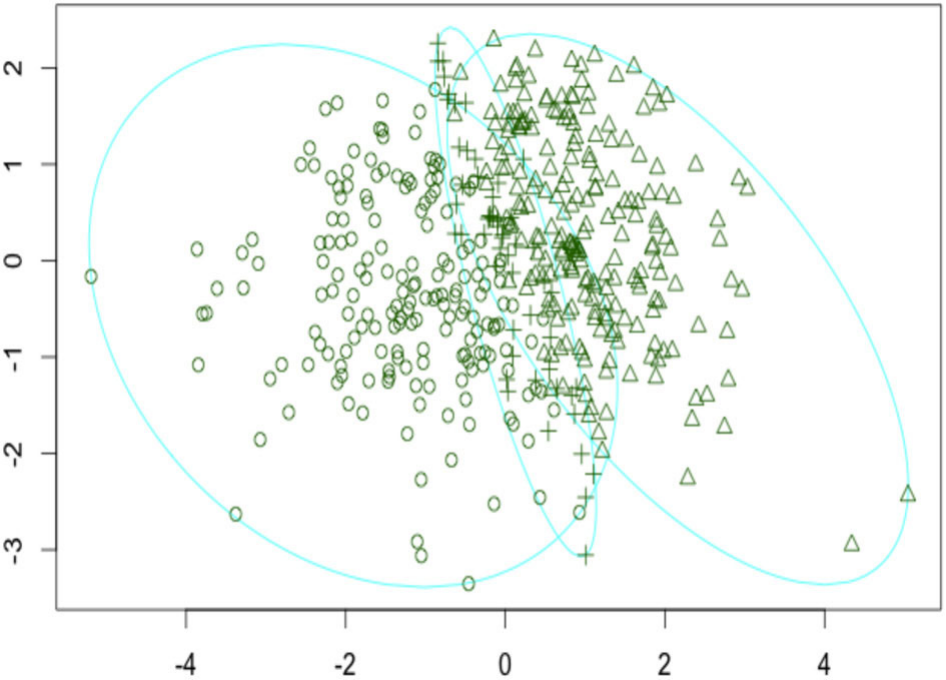
**Visual representation of the 3 cluster fuzzy clustering solution**. Axes are standardized representations of the principal components of the cluster solution.

Thinking about the big picture provided by the 4 cluster fuzzy solution, although the clusters roughly follow the same pattern of means as the K-means solution, it is evident that fuzzy clusters 3 and 4 are very similar indicating that possibly one of the clusters is redundant. This prompted investigation into a 3 cluster fuzzy clustering solution shown in Table 2 and depicted in Figure 1. Looking at the 3 cluster fuzzy clustering solution it seems that fuzzy cluster 3 is very similar in interpretation to clusters 3 and 4 of the 4 cluster fuzzy clustering solution. The remaining two clusters of the 3 cluster fuzzy solution appear to map onto the K-means clusters 1 and 2.

Although Table 2 indicates a similar interpretation for the K-means and fuzzy clustering solutions, the cluster similarity is not absolute. Table 3 presents the percentage of overlap between the four K-means clusters and their corresponding fuzzy clusters. As can be seen, in general each K-means cluster has a clear match to a fuzzy cluster with which it shares a majority of cases. However, clusters 1 and 3 do not map onto the K-means clusters as cleanly. Regarding K-means cluster 1, the highest correspondence can be seen with fuzzy cluster 1, however, they only share 55.4% of their cases. K-means cluster 1 also shares 16.7% of its cases with fuzzy cluster 2 and 26.4% of its cases with fuzzy cluster 3. K-means Cluster 3 shows even less consistency with 44.5% of its cases shared fuzzy cluster 3 and 42.4% of its cases shared with fuzzy cluster 1. An additional 19.5% of its cases are shared with fuzzy cluster 4.

In summary, the 4 cluster solutions obtained from the K-means and the fuzzy clustering methods were similar in interpretation. However, when a moderate degree of cluster overlap was modeled into the clusters (as is the default in fuzzy clustering) two of the clusters appeared nearly indistinguishable to the point that a 3-cluster solution gave nearly the same information. This finding is emphasized in Table 3 with the considerable overlap of fuzzy cluster 3 with multiple K-means clusters. Conceptually speaking, this speaks to potential group similarity of the individuals in these clusters.

### Fuzzy cluster membership

Focusing more fully on the fuzzy clustering solution, relationships between the fuzzy clusters can be investigated by looking at the cluster membership. In fuzzy clustering, cluster membership refers to the degree to which a fuzzy cluster overlaps with another fuzzy cluster. The cluster membership of the 4 cluster fuzzy solution appears in Table 4. According to Table 4, as would be expected, the individuals in each fuzzy cluster belong most strongly to their own cluster than to any other cluster. Consistent with the findings from above, fuzzy clusters 2 and 4 appear to be more distinct than clusters 1 and 3 belonging to their own clusters more strongly (72.6% for cluster 2 and 73.5% for cluster 4) than fuzzy clusters 1 and 3 (67.4% for cluster 1 and 66.5% for cluster 3).

**Table 4:**
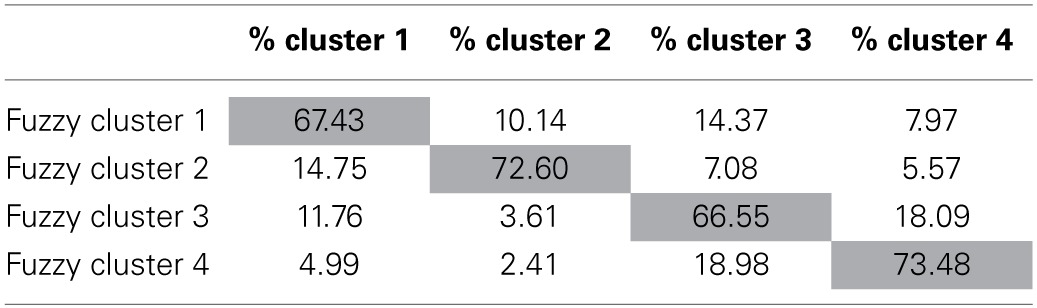
**Summary of clustering membership in percentage for fuzzy clustering**.

Membership Exponent = 2.0

Further information can be gained by examining which clusters overlap. Focusing on fuzzy cluster 4, for example, we can see that there is 18% overlap with fuzzy cluster 3, indicating that, individuals identified with this profile are also similar in characteristics to individuals in cluster 3. Thinking theoretically about these results, conceptual similarities can be seen between clusters 3 (most closely mapping to Internalized Perfectionists) and 4 (most closely mapping to Non-Perfectionists). Each of these clusters demonstrates lower levels on all of the Harvey factor representation for the FMPS (See Table 2). Thus, the conceptual relationship between fuzzy clusters 3 and 4, can be seen through both the cluster membership and the cluster means. Along the same lines, fuzzy cluster 4, shows no practical similarities with fuzzy clusters 1 (most closely mapping to externalized perfectionists) and 2 (most closely mapping to mixed perfectionists) as evidenced by both the small percentages of overlap in the cluster membership (4.99% with fuzzy cluster 1 and 2.41% with fuzzy cluster 2) and the fuzzy cluster means in Table 2.

### Manipulation of cluster overlap

As previously mentioned, the membership exponent used in fuzzy clustering can be changed to increase or decrease the preferred amount of cluster overlap in order to model the hypothesized amount of cluster overlap. In order to investigate the impact of the membership exponent on fuzzy clustering solution, a comparison of the K-means cluster and fuzzy cluster solutions with a membership exponent of 1.2 was compared. The purpose of using a membership exponent of 1.2 is because it allows for less overlap in the clusters thus producing crisper clusters similar to the K-means results while still allowing for fuzziness within the clusters.

Results of this comparison appear in Tables 2, 5. Whereas previously, there were differences between the K-means and fuzzy clusters, when the membership exponent was decreased it created crisper clusters with nearly identical results to the K-means solution. The cluster means for the *ME* = 1.2 solution shown in Table 2 are nearly identical to their K-means cluster counterparts and the cluster correspondence shown in Table 5 is more than 90% for all four clusters indicating strong agreement between the K-means and ME 1.2 fuzzy clustering solutions. Fuzzy clusters 2 and 4, which were already identified as corresponding reasonably well with the K-means solution when the membership exponent was 2.0 are nearly identical (99–100% agreement) to the K-means solution once the membership exponent is set to 1.2. Fuzzy clusters 1 and 3 which exhibited a large degree of overlap with the other clusters in the ME 2.0 solution are also nearly identical to their K-means cluster counterparts with 93.6% agreement for cluster 1 and 97.5% agreement for cluster 3. Thus, the manipulation of the membership exponent to model more distinct clusters with little cluster overlap resulted in a solution nearly identical to the original K-means solution.

**Table 5:**
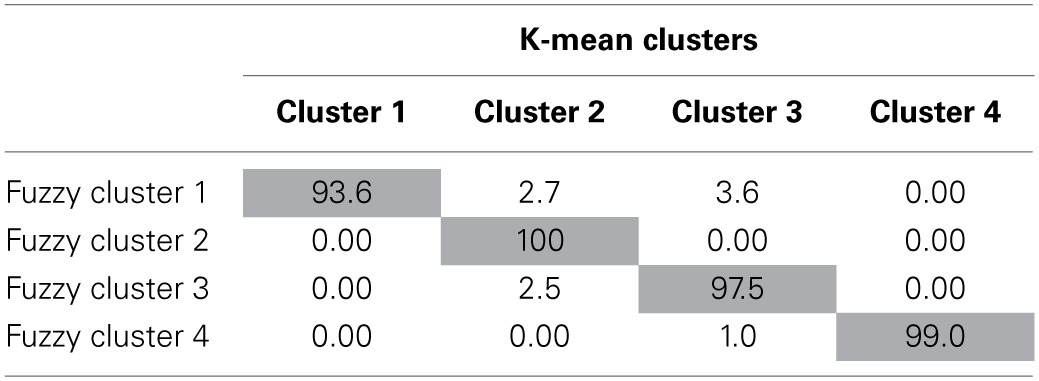
**Percentage of fuzzy cluster solutions that belong to corresponding k-means clustering solutions with a membership exponent of 1.2**.

## Discussion

While hard clustering methods are dominant in the behavioral sciences, there is also great worth in investigating the utility of more flexible clustering algorithms. Fuzzy clustering provides one such technique as it provides more flexibility in the modeling and interpretation of cluster solutions. This study demonstrated that fuzzy clustering is also able to show a different perspective to the cluster solutions, perhaps, better illuminating the nature of relationships between clusters.

Through the first comparison of the four-cluster K-means and fuzzy solutions, we found two unique yet similar cluster solutions. The K-means cluster solution created four distinct, non-overlapping clusters, whereas, fuzzy clustering created two clearly distinct clusters and two clearly overlapping clusters. One potential reason for the difference in cluster solutions between the two methods is due to the way fuzzy clustering handles ambiguity in clusters. Unlike K-means clustering, fuzzy clustering allows observations to belong to multiple clusters, with the primary cluster being the one for which the individual has the largest membership coefficient. In this study, the fuzzy clustering solution included one cluster with moderate means on all factors, one cluster with higher means on all factor, especially negative projections, and two clusters with low means on all factors. This will essentially create a different, yet meaningful alternative solution to that produced by K-means.

The allowance for overlap among the clusters increases the potential utility of fuzzy clustering for gaining insights into the nature of the subgroups present in the population, by demonstrating more clearly than do K-means solutions the proximity and interrelatedness of these groups. From the overlapping clusters in not only the current study, but in other studies as well (e.g., Hwang and Thill, 2009; Ghosh et al., 2011), fuzzy clustering assisted in the understanding of the population structure, and the similarities of subgroups therein. This is in part due to the flexibility in interpreting the clusters and varying degrees of membership that can be shown in fuzzy clustering (Díaz et al., 2006; Coppi et al., 2010). Like most areas in psychology, perfectionism profiles can be seen as abstract since the profiles will naturally have similar attributes in certain areas. Without the use of fuzzy clustering we could only speculate how the means impact the relationship between the clusters. Fuzzy clustering enabled us and other studies (i.e., Grubesic, 2006; Andrews and Beynon, 2011) to appropriately handle overlapping characteristics, relate objects to more than one cluster, and provide more information about the structures of the clusters.

In addition to demonstrating the potential for finding different clustering solutions using the K-means and fuzzy approaches, this study also showed how similar results can also be identified by the two clustering methods through manipulation of the membership exponent in the fuzzy clustering algorithm. When using a very low membership exponent (1.2) the fuzzy algorithm yielded nearly the same cluster solution as did K-means. However, because the membership exponent can be adjusted, thereby changing the degree of overlap allowed between clusters, fuzzy clustering has the added advantage of being able to investigate relationships among clusters (Schreer et al., 1998), as well. This is particularly useful in the behavioral sciences, as not all data situations will have the same degree of ambiguity.

Similar to research on perfectionism, there are other areas in psychology and the social sciences where modeling of overlapping and ambiguous concepts could be beneficial. Chapman and Goldberg's (2011) research on personality provide another example. They point to graphical representations suggesting that their cluster structures were “fuzzy.” Consequently, fuzzy clustering may be thought of as a more natural approach to clustering such data, as it does not force indistinguishable cases into one cluster or another and would be able to model such cluster “fuzziness.” However, despite the fact researchers are aware that not all clusters are discrete, fuzzy clustering has not been utilized to its full potential in the social and behavioral sciences.

### Limitations and directions for future research

The aim of this study was to highlight the advantages of a relatively underutilized and potentially useful form of clustering in the context of the social sciences. It is important to note, however, that the present illustration used the default R settings when running these analyses. Although the default settings do have support as reasonably robust choices, there are many customizations and choices involved in the clustering process, which can impact the final cluster result. Such choices include variables to cluster on and number of clusters as well as technical clustering details such as clustering algorithm, initialization method and dissimilarity measure. See (Kaufman and Rousseeuw, 2005; Everitt et al., 2011) for further details regarding K-means and fuzzy clustering options. Also, like all statistical methods, cluster analysis algorithms can perform poorly when non-optimal data situations arise, such as the presence of outliers, multicollinearity among the variables used to group individuals in the sample, or high skewness of the clustering variables (Everitt et al., 2011). Additionally, due to the maximum likelihood estimation method used in estimation of cluster solutions, optimal solution choice and issues with local maxima do arise, making the choice of initial values for the algorithm very important (Steinley, 2003). Thus, just like any statistical method it is important to consider customization options and potential issues when interpreting cluster solutions.

The purpose of this paper is to provide an illustration of the utility of fuzzy clustering to a research question from the social sciences. Although this illustration brings up many noteworthy points, there is still much that is unknown about the optimal use of fuzzy clustering in practice. For example, there has been little research into optimal usage and accuracy under typical conditions encountered in the social sciences. There is also little research providing advice into choice of membership exponent. In addition, it is not yet known how the performances of fuzzy and K-means clustering compare under a wide variety of data distribution conditions, particularly with respect to classification accuracy, and identification of relationships among clusters. All of these are topics worthy of future research endeavors. It is hoped, however, that this study has introduced social scientists to a clustering technique that can enhance psychological research involving the identification of subgroups in the population while simultaneously laying the foundation for future studies focusing on the benefits, optimal usage, and properties of fuzzy clustering in psychology.

### Conflict of interest statement

The authors declare that the research was conducted in the absence of any commercial or financial relationships that could be construed as a potential conflict of interest.

## References

[B1] Ahmed M. N. Yamany S. M. Mohamed N. Farag A. A. Moriarty T. (2002). A modified fuzzy c-means algorithm for bias field estimation and segmentation of MRI data. IEEE Trans. Med. Imaging 21, 193–199 10.1109/42.996338 11989844

[B2] Alshalalfah M. Alhajj R. (2009). Cancer class prediction: two stage clustering approach to identify information genes. Intell. Data Anal. 13, 671–686 10.3233/IDA-2009-0386

[B3] Andrews R. Beynon M. J. (2011). Organizational form and strategic alignment in a local authority: a preliminary exploration using fuzzy clustering. Public Organ. Rev. 11, 201–218 10.1007/s11115-010-0117-4

[B4] Ashby J. S. Bruner L. P. (2005). Multidimensional perfectionism and obsessive compulsive behaviors. J. College Couns. 8, 31–40 10.1002/j.2161-1882.2005.tb00070.x

[B5] Chapman B. P. Goldberg L. R. (2011). Replicability and 40- year predictive power of childhood ARC types. J. Pers. Soc. Psychol. 101, 593–606 10.1037/a0024289 21744975 PMC3160513

[B6] Coppi R. D'Urso S. Giordani P. (2010). A fuzzy clustering model for multivariate spatial time series. J. Classif. 27, 54–88 10.1007/s00357-010-9043-y

[B7] De Andrés J. Lorca P. Javier de Cos Juez F. Sánchez-Lasheras F. (2011). Bankruptcy forecasting: a hybrid approach using fuzzy c-means clustering and multivariate adaptive regression splines (MARS). Expert Syst. Appl. 38, 1866–1875 10.1016/j.eswa.2010.07.117

[B8] Díaz B. Moniche L. Motillas A. (2006). A fuzzy clustering approach to the key sectors of the Spanish economy. Econ. Syst. Res. 18, 299–318 10.1080/09535310600844375

[B9] Everitt B. Landau S. Leese M. Stahl D. (2011). Cluster Analysis. Chichester: Wiley

[B10] Frost R. O. Marten C. M. Lahart C. Rosenblate R. (1990). The dimensions of perfectionism. Cogn. Ther. Res. 14, 449–468 10.1007/BF01172967

[B13] Gan G. Ma C. Wu J. (2007). Data Clustering: Theory, Algorithms, and Applications. Philadelphia, PA: SIAM, Society for Industrial and Applied Mathematics

[B11] Gaudreau P. (2013). The 2 × 2 model of perfectionism: commenting the critical comments and suggestions of Stoeber (2012). Pers. Individ. Differ. 55, 351–355 10.1016/j.paid.2013.03.021

[B12] Gaudreau P. Thompson A. (2010). Testing a 2X2 model of dispositional perfectionism. Pers. Individ. Differ. 48, 532–537 10.1016/j.paid.2009.11.031

[B14] Ghosh A. Mishra N. S. Ghosh S. (2011). Fuzzy clustering algorithms for unsupervised change detection in remote sensing images. Inf. Sci. 181, 699–715 10.1016/j.ins.2010.10.016

[B15] Gilman R. Ashby J. S. Sverko D. Florell D. Varjas K. (2005). The relationship between perfectionism and multidimensional life satisfaction among Croatian and American youth. Pers. Individ. Differ. 39, 155–166 10.1016/j.paid.2004.12.014

[B16] Goktepe A. B. Altun S. Sezer A. (2005). Soil clustering by fuzzy c-means algorithm. Adv. Eng. Softw. 36, 691–698 10.1016/j.advengsoft.2005.01.008

[B17] Grubesic T. H. (2006). On the application of fuzzy clustering for crime hot spot detection. J. Quant. Criminol. 22, 77–105 10.1007/s10940-005-9003-6

[B18] Grzegorek J. L. Slaney R. B. Franze S. Rice K. G. (2004). Self-criticism, dependency, self-esteem, and grade point average satisfaction among clusters of perfectionists and nonperfectionists. J. Couns. Psychol. 51, 192–200 10.1037/0022-0167.51.2.192

[B19] Guldemir H. Sengur A. (2006). Comparison of clustering algorithms for analog modulation classification. Expert Syst. Appl. 30, 642–649 10.1016/j.eswa.2005.07.014

[B20] Hamachek D. E. (1978). Psychodynamics of normal and neuroticperfectionism. Psychol. J. Hum. Behav. 15, 27–33

[B21] Hartigan J. A. Wong M. A. (1979). Algorithm AS 136: a K-means clustering algorithm. J. R. Stat. Soc. C 28, 100–108

[B22] Harvey B. Pallant J. Harvey D. (2004). An evaluation of the factor structure of the Frost Multidimensional Perfectionism Scale. Educ. Psychol. Measur. 64, 1007–1018 10.1177/0013164404264842

[B23] Hewitt P.L. Flett G. L. (1991). Perfectionism in the self and social contexts: conceptualization, assessment, and association with psychopathology. J. Pers. Soc. Psychol. 60, 456–470 10.1037//0022-3514.60.3.456 2027080

[B24] Hu B. G. Gosine R. G. Cao L. X. de Silva C. W. (1998). Application of a fuzzy classification technique in computer grading of fish products. IEEE Trans. Fuzzy Syst. 6, 144–152 10.1109/91.660814

[B25] Hwang S. Thill S-C. (2009). Delineating urban housing submarkets with fuzzy clustering. Environ. Plann. B Plann. Des. 36, 865–882 10.1068/b34111t

[B26] Izenman A. J. (2008). Modern Multivariate Statistical Techniques: Regression Classification And Manifold Learning. New York, NY: Springer Science+Business Media LLC

[B27a] Kaufman L. Rousseeuw P. J. (1990). Finding Groups in Data: An Introduction to Cluster Analysis. New York, NY: John Wiley and Sons

[B27] Kaufman L. Rousseeuw P. J. (2005). Finding Groups in Data: An Introduction to Cluster Analysis. Hoboken, NJ: Wiley

[B28] Lekova A. (2010). Evolving fuzzy modeling for MANETs using lightweight online unsupervised learning. Int. J. Wireless Inf. Netw. 17, 34–41 10.1007/s10776-010-0114-0

[B29] Maharaj E. A. D'Urso P. (2011). Fuzzy clustering of time series in the frequency domain. Inf. Sci. 181, 1187–1211 10.1016/j.ins.2010.11.031

[B30] Mobley M. Slaney R. B. Rice K. G. (2005). Cultural validity of the almost perfect scale—revised for African American college students. J. Couns. Psychol. 52, 629–639 10.1037/0022-0167.52.4.629

[B31] Ooi W. S. Lim C. P. (2006). Fuzzy clustering of color and texture features for image segmentation: a study on satellite image retrieval. J. Intell. Fuzzy Syst. 17, 297–311

[B32] Parker W. D. (1997). An empirical typology of perfectionism in academically talented children. Am. Educ. Res. J. 34, 545–562 10.3102/00028312034003545

[B33] Purdon C. Antony M. M. Swinson R. P. (1999). Psychometric properties of the Frost Multidimensional Perfectionism Scale in a clinical anxiety disorders sample. J. Clin. Psychol. 55, 1271–1286 10.1002/(SICI)1097-4679(199910)55:10<1271::AID-JCLP8>3.0.CO;2-A 11045776

[B34] R Development Core Team (2010). R: A Language and Environment for Statistical Computing. Vienna: R Foundation for Statistical Computing

[B35] Rice K. G. Dellwo J. P. (2002). Perfectionism and self-development: implications for college adjustment. J. Couns. Dev. 80, 188–196 10.1002/j.1556-6678.2002.tb00182.x

[B36] Schreer J. F. O'Hara Hines R. J. Kovacs K. M. (1998). Classification of dive profiles: a comparison of statistical clustering techniques and unsupervised artificial neural networks. J. Agric. Biol. Environ. Stat. 3, 383–404

[B37] Slaney R. B. Rice K. G. Mobley M. Trippi J. Ashby J. S. (2001). The revised almost perfect scale. Meas. Eval. Couns. Dev. 34, 130–145

[B38] Steinley D. (2003). Local optima in K-means clustering: what you don't know may hurt you. Psychol. Methods 8, 294–304 10.1037/1082-989X.8.3.294 14596492

[B39] Stoeber J. (1998). The Frost MPS revisited: more perfect with four (instead of six) dimensions. Pers. Individ. Differ. 24, 481–491 10.1016/S0191-8869(97)00207-9

[B40] Stoeber J. Feast A. R. Hayward J. A. (2009). Self-oriented and socially prescribed perfectionism: differential relationships with intrinsic and extrinsic motivation and test anxiety. Pers. Individ. Differ. 47, 423–428 10.1016/j.paid.2009.04.014

[B41] Stoeber J. Otto K. (2006). Positive conceptions of perfectionism: approaches, evidence, and challenges. Pers. Soc. Psychol. Rev. 10, 295–319 10.1207/s15327957pspr1004_2 17201590

[B42] Wang J. Bø T. H. Jonassen I. Myklebost O. Hovig E. (2003). Tumor classification and marker gene prediction by feature selection and fuzzy c-means clustering using microarray data. BMC Bioinformatics 4:60 10.1186/1471-2105-4-60 14651757 PMC302113

